# The Roles of the Paf1 Complex and Associated Histone Modifications in Regulating Gene Expression

**DOI:** 10.4061/2011/707641

**Published:** 2011-11-17

**Authors:** Elia M. Crisucci, Karen M. Arndt

**Affiliations:** Department of Biological Sciences, University of Pittsburgh, Pittsburgh, PA 15260, USA

## Abstract

The conserved Paf1 complex (Paf1C) carries out multiple functions during transcription by RNA polymerase (pol) II, and these functions are required for the proper expression of numerous genes in yeast and metazoans. In the elongation stage of the transcription cycle, the Paf1C associates with RNA pol II, interacts with other transcription elongation factors, and facilitates modifications to the chromatin template. At the end of elongation, the Paf1C plays an important role in the termination of RNA pol II transcripts and the recruitment of proteins required for proper RNA 3′ end formation. Significantly, defects in the Paf1C are associated with several human diseases. In this paper, we summarize current knowledge on the roles of the Paf1C in RNA pol II transcription.

## 1. Introduction

The RNA pol II transcription cycle can be divided into three primary stages: initiation, elongation, and termination. During transcription initiation, the binding of the TATA-binding protein (TBP) subunit of TFIID to the promoter triggers the assembly of a preinitiation complex, which contains RNA pol II and the general transcription factors, TFIIA, TFIIB, TFIID, TFIIE, TFIIF, and TFIIH (reviewed in [1]). The general transcription factors position the polymerase at the transcription start site and unwind the DNA to expose the template strand for RNA synthesis (reviewed in [1]). During transcription elongation, multiple regulatory proteins associate with RNA pol II to facilitate its progression and modify the chromatin template (reviewed in [2]). Finally, during RNA 3′ end formation and transcription termination, the transcript is processed and released through the combined actions of multiple RNA processing factors (reviewed in [3]). Therefore, each stage of the transcription cycle is regulated by a plethora of proteins to ensure proper gene expression. 

The regulation of transcription initiation is an important aspect of controlling gene expression and has thus been studied for many years. More recently, the regulation of postinitiation stages has been shown to be equally important for ensuring proper gene expression. Furthermore, eukaryotic cells have evolved many mechanisms to overcome the barriers imposed by chromatin on all three stages of the transcription cycle. One highly conserved protein complex that lies at the intersection between chromatin modification pathways and transcription is the Paf1C. This complex associates with RNA pol II [4–7] and influences multiple events during transcription elongation, including the posttranslational modification of histone proteins [8–12] and the recruitment of proteins required for RNA processing [13–16]. Not surprisingly given its key regulatory roles, the Paf1C and its functions are conserved throughout eukaryotes. Here, we review current knowledge on the Paf1C, emphasizing insights that have emerged from genetic and biochemical studies in budding yeast and also discussing more recent observations made in multicellular eukaryotes, where defects in the complex lead to developmental abnormalities and disease.

## 2. Initial Studies in Yeast Led to the Discovery of the Paf1C and Revealed Its Roles in Transcription Elongation

The search for accessory proteins that cooperate with general transcription factors and regulate transcription initiation prompted experiments that led to the identification of Paf1 (polymerase-associated factor 1) in *Saccharomyces cerevisiae* [7, 17]. As the name implies, Paf1 was found to associate with RNA pol II by affinity chromatography [17]. Subsequent studies demonstrated that Paf1 exists in a nuclear complex with Ctr9, Cdc73, Rtf1, and Leo1 [4, 18–20]. Prior to the discovery that they interacted with Paf1, other members of the Paf1C were identified through yeast genetics and recognized for their potential roles in transcription. For example, the *CTR9* gene (Cln three (*CLN3*) requiring 9) was originally identified by its genetic interaction with *CLN3* [21] and subsequently identified in a genetic screen for mutants with impaired transcription of G1 cyclin genes [22]. Additionally, *RTF1* (Restores TBP function 1) was originally discovered in a genetic selection for mutations that suppress transcriptional defects caused by TBP mutants with altered DNA binding specificity [23]. Cdc73 (Cell division cycle 73) [24] and Leo1 (Left open reading frame 1) [25] may not have been initially recognized for their roles in transcription, but were subsequently determined to be important transcriptional regulators in the context of the Paf1C. 

**Figure 1:**
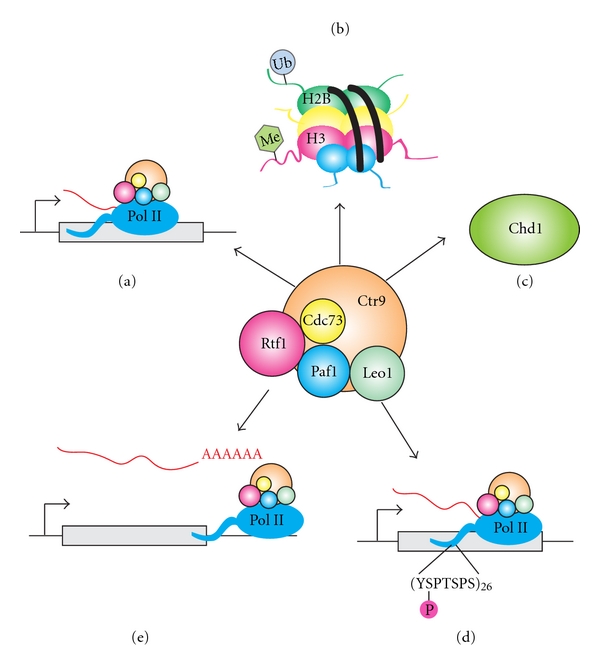
The multiple functions of the Paf1C. During transcription elongation, the Paf1C (a) associates with RNA pol II on coding regions [4, 6], (b) regulates histone modifications [8–12] (discussed in detail in Figure 2), and (c) recruits Chd1, an ATP-dependent chromatin remodeling enzyme [131, 132]. (d) During a later stage of transcription elongation, the Paf1C promotes phosphorylation of serine 2 of the RNA pol II CTD [13, 31]. (e) Additionally, the Paf1C is important for proper transcription termination and RNA 3′ end formation of both polyadenylated and nonpolyadenylated transcripts [13–16, 31].

Paf1C subunits have been implicated in transcription initiation by influencing the phenotypic effects of a TBP-altered specificity mutant [23] and in transcription termination and RNA 3′ end formation by mediating recruitment of 3′ end processing factors [13–16]. However, the Paf1C is currently best characterized for its critical roles during transcription elongation. Initial studies revealed that genetic disruption of the yeast Paf1C causes phenotypes associated with transcription elongation defects. For example, *S. cerevisiae* strains lacking Paf1C subunits exhibit sensitivity to 6-azauracil (6-AU) and mycophenolic acid (MPA) [20, 26]. These drugs reduce intracellular nucleotide pools, which is thought to increase polymerase pausing, making transcription more dependent on regulatory factors [27]. Consistent with these phenotypes, Paf1C members genetically and physically interact with elongation factors such as the Spt4-Spt5 (yDSIF) and Spt16-Pob3 (yFACT) complexes, suggesting that these complexes function cooperatively to modulate transcription elongation [4, 20, 26]. In agreement with the genetic data, a recently described transcription run-on assay revealed transcription elongation defects in the absence of Paf1C subunits *in vivo* [28]. Despite the strong evidence currently linking the Paf1C to the control of transcription elongation, a less direct role in regulating gene expression was also proposed in an earlier study [29]. The lack of an effect of *rtf1*Δ and *cdc73*Δ mutations on *in vivo* elongation rates or RNA pol II processivity, as measured on an inducible long gene, led to the conclusion that the Paf1C influenced cotranscriptional processes. Indeed, the Paf1C has been implicated in several cotranscriptional processes, including the phosphorylation of RNA pol II during elongation and the recruitment of a chromatin-remodeling enzyme, Chd1, to open reading frames [13, 30, 31]. Additionally, in its best-understood role, the Paf1C is important for the establishment of cotranscriptional histone modifications that influence gene expression [8–12]. Together, these observations suggest that the Paf1C influences gene expression through multiple functions during transcription (Figure 1). In this paper, we describe current information on these functions. 

## 3. The Paf1C Associates with RNA Pol II and Influences the Phosphorylation State of the RNA Pol II CTD

The Paf1C accompanies the polymerase from the transcription start site to the poly(A) site [5, 32]. Rtf1 and Cdc73 are both required for the physical association of Paf1C with RNA pol II. In *rtf1*Δ or *cdc73*Δ cells, the remaining Paf1C subunits dissociate from the polymerase and chromatin, even though these subunits remain associated in a subcomplex [13, 31, 33]. Deletion analysis of *S. cerevisiae RTF1* defined a central region of the Rtf1 protein (amino acids 201 to 395), termed the ORF association region (OAR), that is required for the physical association between the Paf1C and active genes [34]. Although an NMR study has provided important structural information on the human Rtf1 OAR, also known as a Plus3 domain [35], the manner in which Rtf1 interacts with RNA pol II is unknown. Recombinant Cdc73 can interact with purified RNA pol II, suggesting that Cdc73 may directly contact RNA pol II *in vivo* [19]. Beyond the interactions of Rtf1 and Cdc73 with RNA pol II, Leo1 is also required for full association of the Paf1C with active genes [36]. In this case, evidence suggests that an interaction between Leo1 and the nascent mRNA stabilizes the association of the Paf1C with transcribed genes [36].

The interaction between Paf1C subunits and elongating RNA pol II is modulated by other transcription elongation factors. Several reports demonstrated that the Spt4-Spt5 complex promotes recruitment of the Paf1C to chromatin [33, 37–39]. Interestingly, recent studies suggest that the functional interactions between the Paf1C, Spt4-Spt5, and RNA polymerase are conserved beyond RNA pol II and are important for RNA pol I transcription as well [40–44]. Although their roles in Paf1C recruitment are less well characterized than that of Spt4-Spt5, the Spt6, FACT, and Ccr4-Not transcription factors have also been shown to modulate recruitment of the Paf1C to active genes [45–47]. 

The C-terminal domain (CTD) of the largest subunit of RNA pol II, Rpb1, consists of tandemly repeated copies of a heptapeptide sequence (YSPTSPS) that can be phosphorylated on the serines at positions 2, 5, and 7 of the repeat. Importantly, the phosphorylation state of the CTD changes throughout the transcription cycle and is important for recruiting the appropriate regulatory factors during each stage of transcription (reviewed in [48]). During initiation, the RNA pol II CTD is hypophosphorylated. Upon the transition from initiation to early elongation, the CTD becomes phosphorylated on serine 5 by CDK7 (Kin28 in yeast) of the general transcription factor TFIIH [49]. Phosphorylated serine 5 is recognized by the mRNA capping machinery, thus coordinating mRNA 5′ end capping and early transcription elongation [50]. In yeast, phosphorylation of serine 5 can be reversed by Ssu72 and tends to decline as elongation proceeds [51]. Serine 7 of the CTD repeats is also phosphorylated by Kin28 [52–54]. Patterns of serine 5 and 7 phosphorylation overlap across genes; however, the elucidation of the functions of serine 7 phosphorylation is still at an early stage [52–55]. Later in elongation, serine 2 of the CTD becomes phosphorylated mainly by Ctk1 in yeast or P-TEFb in human cells [56, 57]. Serine 2 phosphorylation promotes the recruitment of cleavage and polyadenylation factors to RNA pol II, connecting the later stages of elongation to RNA 3′ end processing [58, 59]. Through mechanisms that are undefined, the Paf1C is required for normal levels of serine 2 phosphorylation [13, 31]. In addition to termination and RNA 3′ end formation factors, serine 2 phosphorylation recruits the histone H3 lysine (K) 36 methyltransferase, Set2 [60–63]. Therefore, the Paf1C most likely impacts these processes, in part, through influencing CTD phosphorylation.

## 4. The Paf1C Influences Gene Expression by Promoting Histone H2B K123 Ubiquitylation and Histone H3 K4 and K79 Methylation

During transcription elongation, RNA pol II encounters obstacles in the form of nucleosomes, the basic units of chromatin. Nucleosomes consist of two copies of each of the four histone proteins, H2A, H2B, H3, and H4, in a globular arrangement, wrapped by 147 base pairs of DNA [64, 65]. A large amount of evidence indicates that nucleosomes impede transcription elongation. For example, elongation efficiency is severely reduced during transcription of reconstituted chromatin templates compared to naked DNA *in vitro* [66, 67]. Furthermore, *in vivo*, transcription rates inversely correlate with nucleosome occupancy within open reading frames (ORFs) [68]. In a recent study that employed a deep-sequencing-based method to determine the positions of all active RNA pol II molecules, extensive pausing and backtracking of the polymerase were observed throughout the bodies of genes [69]. Paused polymerase was particularly noticeable at the positions of the first four nucleosomes, confirming that nucleosomes act as a barrier to transcription elongation *in vivo*.

**Figure 2:**
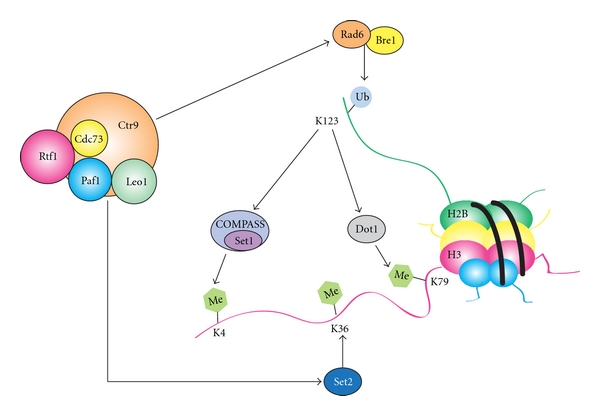
The Paf1C promotes histone H2B monoubiquitylation and histone H3 K4, K36, and K79 methylation. In yeast, the ubiquitin conjugating enzyme, Rad6, and the ubiquitin ligase, Bre1, monoubiquitylate H2B K123 [179–181]. H2B monoubiquitylation is a prerequisite for di- and trimethylation of H3 K4 and K79 by the histone methyltransferases Set1 and Dot1, respectively [71, 80, 182, 183]. Histone H3 is methylated on K36 by the methyltransferase Set2 [184]. Paf1 and Rtf1 subunits of the Paf1C are required for H2B K123 monoubiquitylation and the downstream di- and trimethylation of H3 K4 and K79 [9–12]. Paf1 and Ctr9 are required for trimethylation of K36 on histone H3 [8].

Histone proteins are subject to a wide variety of posttranslational modifications, including the acetylation, ubiquitylation, and methylation of lysine residues. These modifications regulate RNA pol II activity at all stages of the transcription cycle. Members of the Paf1C are required for several histone modifications that are associated with active transcription (Figure 2). Specifically, Paf1 and Rtf1 are required for the monoubiquitylation of H2B on K123 in yeast (K120 in humans) [12, 37, 70] and the subsequent di- and trimethylation of H3 K4 and K79 [9–11, 71]. Paf1 and Rtf1 promote H2B monoubiquitylation by facilitating the association of Rad6 with RNA pol II during transcription elongation [9–12, 70]. An *in vitro *assay using purified factors revealed a direct interaction between the Paf1C and Bre1 [72]; therefore, the Paf1C may tether Rad6 and Bre1 to the elongating polymerase. Since *paf1*Δ cells have greatly reduced levels of Rtf1 protein, Rtf1 is probably the primary subunit that promotes H2B monoubiquitylation and subsequent methylation of H3 on K4 and K79 [13]. In fact, mutational studies have shown that amino acids 62–152 of *S. cerevisiae* Rtf1 are required for these histone modifications, leading to the definition of a histone modification domain (HMD) in Rtf1 [34, 73]. 

H2B K123 ubiquitylation and H3 K4 and K79 methylation are enriched on the coding regions of active genes [70, 74, 75]. Consistent with a positive role in transcription, H2B monoubiquitylation has been shown to enhance the transcription elongation rate of a chromatin template *in vitro* [47]. *In vivo*, H2B K123 ubiquitylation facilitates transcription of galactose-inducible genes in yeast by promoting nucleosome-reassembly in the wake of RNA pol II in cooperation with the histone chaperone, Spt16 [76]. Additionally, a recent study using chemically defined nucleosome arrays demonstrated that H2B ubiquitylation interferes with chromatin compaction, which may facilitate transcription [77]. 

H2B K123 ubiquitylation and the downstream methylation of H3 on K4 and K79 regulate the silencing of reporter genes positioned near telomeres and other heterochromatic loci within the yeast genome [10, 78–83]. In *S. cerevisiae*, the silencing of genes near telomeres, and at the *HMR*, *HML*, and rDNA loci, is mediated by silent information regulator (Sir) proteins, which preferentially bind to hypomethylated histones (reviewed in [84]). The genome-wide loss of H3 K4 and K79 methylation has been proposed to cause a redistribution of Sir proteins from the silent loci, resulting in the loss of Sir-dependent transcriptional silencing (reviewed in [85]). Paf1 and Rtf1 are required for silencing of a telomere-proximal reporter gene in yeast [9, 10, 34, 73]. However, recent studies indicate that caution should be exercised in generalizing results obtained with the widely used *URA3*-based silencing reporter assays. In these studies, a *dot1*Δ mutation, which has been reported to cause a strong defect in telomeric silencing based on the reporter assays, does not alleviate repression of natural genes near telomeres or lead to global changes in Sir protein occupancy [86, 87]. Based on these new observations, more work will be needed to clarify the roles of the Paf1C and its dependent histone modifications in heterochromatic gene silencing.

Microarray analysis of transcript levels in cells lacking the H2B ubiquitylation site (*htb1-K123R* substitution) has shown that H2B K123 ubiquitylation represses many genes throughout the yeast genome [88]. In fact, the majority of affected genes exhibited increased expression in *htb1-K123R* cells, indicating that this modification predominantly acts to repress transcription [88]. Consistent with repressive functions, reversal of H2B K123 ubiquitylation by the de-ubiquitylating enzyme, Ubp8, is required for full expression of certain inducible genes, including *GAL1*, *GAL10*, and *SUC2 *[89–91]. Furthermore, the Paf1C mediates repression of a subset of genes, including the *ARG1* gene, by facilitating H2B K123 ubiquitylation [92]. These observations suggest that Paf1C-dependent H2B ubiquitylation has important functions for repression of global transcription. The mechanism by which H2B monoubiquitylation represses transcription is not completely understood. However, H2B monoubiquitylation has been shown to increase nucleosome stability at the promoters of repressed genes [93] and antagonize the recruitment of the positive transcription elongation factor TFIIS to genes in human cells [94].

Importantly, like its yeast counterpart, the human Paf1C controls gene expression through H2B monoubiquitylation and H3 K4 and K79 methylation [47, 95–98]. Furthermore, H2B monoubiquitylation in humans also has both positive and negative effects on transcription. For example, H2B monoubiquitylation is preferentially associated with highly expressed genes [97]. In addition, this modification has been shown to stimulate proper *HOX* gene expression in human cells [98] and the transcription of pluripotency genes in embryonic stem cells [95], thus promoting proper development and stem cell identity, respectively. However, de-ubiquitylation of H2B by Usp22, the human homolog of yeast Ubp8, inhibits heterochromatic silencing and promotes gene activation [99, 100]. Human Bre1/RNF20 acts as a tumor suppressor by promoting transcription of tumor suppressor genes and repressing proto-oncogenes, underscoring the importance of both positive and negative gene regulation by H2B monoubiquitylation [101]. Collectively, these observations indicate that H2B monoubiquitylation has important effects on gene expression in both yeast and humans.

## 5. The Paf1C Promotes Histone H3 K36 Trimethylation and Affects Histone Acetylation Levels on Genes

**Figure 3:**
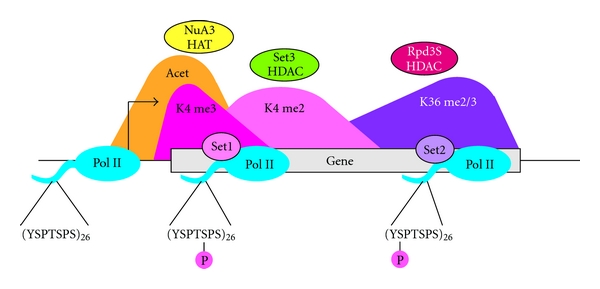
Typical distribution of histone modifications across a gene. Serine 5 phosphorylation on the RNA pol II CTD recruits Set1 to RNA pol II early in elongation, resulting in a peak of H3 K4 trimethylation near promoters [11]. Just downstream, K4 dimethylation peaks in 5′ coding regions [102, 103]. Later in elongation, serine 2 phosphorylation of the RNA pol II CTD recruits Set2, resulting in H3 K36 methylation toward the 3′ end of the coding region [60–63]. H3 K4 trimethylation recruits the NuA3 histone acetyltransferase (HAT) complex, resulting in increased H3 K14 acetylation near the promoter [104, 105]. Within the coding region, dimethylation of H3 K4 promotes the activity of the Set3 histone deacetylase complex (HDAC) [106, 107]. At the 3′ coding region, H3 K36 dimethylation promotes the activity of the Rpd3S HDAC [108–111]. Through these mechanisms, histone acetylation levels are maintained at low levels on coding regions.

In addition to methylation of H3 K4 and K79, Paf1 and Ctr9 are required for trimethylation of H3 K36 by the histone methyltransferase, Set2 [8]. Set2 associates with the elongating form of RNA pol II in the body of actively transcribed genes in a Paf1C-dependent manner [8, 60, 63]. As stated above, the Paf1C may influence Set2 recruitment indirectly through its effects on CTD phosphorylation [13, 31]. Both H3 K4 and K36 methylation occur across most genes in a distinct pattern that is influenced by the phosphorylation state of the RNA pol II CTD (Figure 3). Serine 5 phosphorylation by Kin28 recruits Set1 to RNA pol II early in elongation, resulting in a peak of H3 K4 trimethylation near promoters [11]. Just downstream, K4 dimethylation peaks in 5′ coding regions, whereas K4 monomethylation occurs across the gene [102, 103]. Later in elongation, serine 2 phosphorylation of the RNA pol II CTD recruits Set2, resulting in H3 K36 methylation toward the 3′ end of the coding region [60–63]. 

Interestingly, H3 K4 and K36 methylation modulate histone acetylation by facilitating the recruitment or activity of histone acetyltransferase (HAT) and histone deacetylase complexes (HDACs). H3 K4 trimethylation recruits the NuA3 HAT complex, resulting in increased H3 K14 acetylation [104, 105]. Dimethylation of H3 K4 stimulates the activity of the Set3 HDAC [106, 107]. Consistent with this pathway of H3 K4 methylation-directed deacetylation, the loss of Paf1 results in increased acetylation at 5′ coding regions [8]. Histone H3 K36 dimethylation promotes the activity of the Rpd3S HDAC [108–111]. Through these pathways, H3 methylation restricts histone acetylation to promoters to prevent inappropriate transcription from cryptic start sites internal to coding regions and restore chromatin in the wake of the polymerase. Analysis of *paf1*Δ* set2*Δ double mutant strains suggests that Paf1 and Set2 function separately to reduce cryptic initiation and histone acetylation at 3′ coding regions [8]. These results may not be surprising since Paf1 is selectively required for H3 K36 trimethylation [8], yet dimethylation is sufficient for Rpd3S HDAC activity [111]. Therefore, at 5′ coding regions, the Paf1C reduces histone acetylation, possibly through H3 K4 methylation-mediated deacetylation by Set3. However, at 3′ coding regions, the Paf1C reduces acetylation through an undefined mechanism that is parallel to the established Set2-Rpd3S pathway. 

 Given its important roles in modulating several histone modifications, the Paf1C likely regulates gene expression by promoting histone modifications. However, while genome-wide analysis identified numerous genes that require the Paf1C for proper expression [14], only a subset of Paf1C-responsive genes exhibit altered expression in the absence of these same histone modifications [88]. Therefore, the Paf1C likely has roles aside from facilitating histone modifications that control gene expression. Consistent with this hypothesis, the repressive effect of the yeast Paf1C on *ARG1* transcription can be only partially explained by a loss of histone modifications [92]. Furthermore, *in vitro* transcription elongation assays have revealed a role for the Paf1C in stimulating transcription elongation of naked DNA templates by both RNA pol I and pol II [44, 112]. The histone modification-independent functions of the Paf1C may be conserved throughout eukaryotes, as the human Paf1C has recently been shown to stimulate *in vitro* transcription of a chromatin template independently of histone modifications [113]. Further investigation is required to elucidate critical histone modification-independent functions of the Paf1C.

## 6. The Paf1C Functions Cooperatively with Other Factors That Influence Chromatin Structure

Beyond influencing gene expression through establishing histone modifications, the Paf1C interacts physically and genetically with several factors that influence chromatin structure and transcription elongation, including the elongation complex Spt4-Spt5, the histone chaperone FACT, and the chromatin remodeler Chd1. Spt4-Spt5 is required for the recruitment of the Paf1C to the elongation complex and for H2B K123 ubiquitylation [33, 37–39]. Paf1C recruitment is regulated by phosphorylation of Spt5 by the Bur1 kinase [37–39]. Consistent with cooperative functions, the human Paf1C, Spt4-Spt5/DSIF, and Tat-SF1 cooperatively stimulate transcription elongation *in vitro* and promote transcription *in vivo* [114]. A recent study involving a novel transcription elongation reporter template demonstrated that Spt4 and the Paf1C facilitate elongation in yeast cells [28]. However, biochemical experiments in yeast and human cells have shown that Spt4-Spt5/DSIF interacts with RNA pol II during elongation and has both positive and negative effects on elongation [4, 115–118]. Although the functions of Spt4-Spt5/DSIF are not completely understood, genetic interactions with elongation and chromatin-related factors suggest that Spt4-Spt5/DSIF regulates transcription elongation through the modulation of chromatin structure. For example, in yeast Spt4-Spt5 genetically interacts with the ATP-dependent chromatin remodeler, Chd1 [30, 119], and kinases and phosphatases that modify the CTD of RNA pol II [120]. Spt4-Spt5 has been shown to recruit the Rpd3S HDAC to active genes in cooperation with Kin28 and Ctk1 [121]. Therefore, Spt4-Spt5 may affect chromatin in part by recruiting the Rpd3S HDAC. However, *in vitro *transcription using a naked DNA template has shown that the Paf1C and DSIF can also stimulate transcription elongation independently of chromatin [114]. 

The yeast Paf1C physically and genetically interacts with Spt16-Pob3/FACT [20, 26]. Consistent with this, the human Paf1C augments FACT-stimulated *in vitro* transcription of a chromatin template [47]. FACT was originally identified as a factor that facilitates transcription of a reconstituted chromatin template [67, 122]. Beyond this, many physical and genetic interactions suggest that Spt16-Pob3/FACT has important roles during transcription [20, 26, 30, 122, 123]. It is now known that FACT directly participates in the reorganization of nucleosomes within the ORFs of actively transcribed genes and reassembles chromatin in the wake of RNA pol II [123–127]. *In vitro* binding of an H2A-H2B dimer by FACT has led to the conclusion that FACT displaces a single H2A-H2B dimer to allow RNA pol II passage [122, 128, 129]. However, analysis of nucleosomes by *in vitro* hydroxyl radical accessibility and endonuclease cleavage experiments showed that FACT creates more accessibility than could be explained by the loss of an H2A-H2B dimer, yet partially protects nucleosomal DNA [130]. These results have led to a second model for FACT function in which FACT performs a more dramatic reorganization of the nucleosome without histone eviction. 

In addition to its physical interactions with Spt4-Spt5 and FACT, the Paf1C interacts with Chd1, a conserved ATP-dependent chromatin remodeling enzyme [131, 132]. Chd1 associates with regions of active transcription [30, 133] and physically interacts with Spt4-Spt5/DSIF, FACT, and the Paf1C [4, 30, 134], pointing to an important role during transcription elongation. The mechanistic details of chromatin remodeling by Chd1 are not well understood. However, Chd1 has been shown to create a chromatin structure that inhibits cryptic transcription initiation [119]. Chd1 contains two N-terminal chromodomains, a central ATPase domain and a C-terminal DNA-binding domain [135]. Interestingly, structural studies revealed that the two Chd1 chromodomains regulate the ATPase motor in a unique manner. Specifically, in the absence of nucleosome binding, the chromodomains physically block the ATPase domain, preventing association with naked DNA [136]. Chromodomains also bind methylated lysines [137]. It has been shown that the human homolog of Chd1 associates with chromatin by recognition of H3 K4 methylation [138, 139], but there are differing reports as to whether this occurs in yeast [138–140]. Instead, the Rtf1 subunit of the Paf1C in yeast has been shown to recruit Chd1 to chromatin through a region of Rtf1 distinct from its histone modification domain [30, 34].

## 7. The Paf1C Coordinates Transcription Elongation with Transcription Termination and RNA 3′ End Processing

In addition to its critical functions during transcription elongation, the Paf1C is important for proper transcription termination and RNA 3′ end formation [13–16, 31]. The loss of Paf1C members results in shorter poly(A) tail lengths [13]. Additionally, the Paf1C has been shown to modulate expression of a subset of genes, not by regulating elongation, but by controlling poly(A) site usage [14]. Specifically, the loss of Paf1 results in the read-through of poly(A) sites, producing 3′-extended transcripts that are subject to nonsense-mediated decay [14]. Termination and RNA 3′ end processing defects that occur in the absence of Paf1 can be attributed to the reduced recruitment of 3′ end processing factors to chromatin. In the absence of Paf1C members, altered poly(A) site usage is associated with reduced chromatin association of the cleavage and polyadenylation factor Pcf11 [13]. Additionally, Cft1, another 3′ end processing factor, associates with RNA pol II in a Paf1C-dependent manner [31]. The recruitment of cleavage and polyadenylation factors to RNA pol II and chromatin requires the serine 2-phosphorylated form of the RNA pol II CTD [58, 141]. Therefore, the Paf1C may regulate the recruitment of 3′ end processing factors indirectly through its effects on CTD phosphorylation. However, direct interactions between the Paf1C and 3′ end processing factors have been demonstrated in yeast and humans [31, 142]. Therefore, the Paf1C may recruit 3′ end processing factors through a mechanism that does not rely on RNA pol II CTD phosphorylation. 

Together, these observations suggest that the Paf1C plays an important role in coordinating transcription with RNA 3′ end processing. Given that the Paf1C is required for the recruitment of 3′ end processing factors to chromatin [13, 31], yet it dissociates from RNA pol II shortly after the poly(A) site has been transcribed [5, 32], the Paf1C appears to participate in an exchange of elongation factors for 3′ end processing factors during transcription termination. Consistent with this hypothesis, when dissociated from chromatin, the Paf1C associates with RNA processing factors [31]. However, the exact mechanism by which the Paf1C regulates termination and 3′ end processing of polyadenylated transcripts remains unclear. 

The Paf1C is also required for proper termination and 3′ end formation of nonpolyadenylated transcripts [15]. The loss of Paf1C members or Paf1C-dependent histone modifications results in the synthesis of small nucleolar RNAs (snoRNAs) extended at their 3′ ends [15, 73]. snoRNA termination defects in the absence of Paf1C members are associated with reduced recruitment of the 3′ end processing factors, Nrd1 and Nab3 [15]. Therefore, similar to its effects on the termination of polyadenylated transcripts, the Paf1C mediates snoRNA termination by promoting recruitment of 3′ end processing factors. Interestingly, it has recently been shown that the termination function of the Paf1C can be inhibited through an interaction with an activator [143]. Specifically, a physical interaction between Mpk1 MAPK and Paf1 prevents premature transcription termination by inhibiting recruitment of the Sen1-Nrd1-Nab3 complex [143]. However, the mechanism by which the Paf1C recruits 3′ end processing factors for termination remains to be revealed. Additionally, disruption of the Rtf1 HMD results in snoRNA termination defects, implicating H2B K123 ubiquitylation in the regulation of transcription termination [73]. Interestingly, nucleosome depletion in terminator regions has been shown to require RNA pol II transcription [144]. Therefore, in addition to facilitating recruitment of 3′ end processing factors, the Paf1C promotes proper transcription termination through H2B monoubiquitylation and its effects on chromatin structure. 

The contribution of the Paf1C to transcription termination has yet to be assessed on a genome-wide scale. However, given the essential roles of transcription termination, which include regulating transcript stability and RNA pol II recycling (reviewed in [145–148]), Paf1C-dependent termination is likely to have wide-spread effects on gene expression. Importantly, the functions of the Paf1C in regulating termination and RNA 3′ end formation are conserved from yeast to humans [142, 149].

## 8. The Paf1C Has Critical Functions in Metazoans

As mentioned above, the known functions of the Paf1C, including RNA pol II-association [150] and roles in transcription elongation [113, 114], histone modification [95, 96, 98, 142], and RNA 3′ end formation [142, 149], are conserved between yeast and humans. However, there are some differences in complex composition in yeast and higher eukaryotes. In humans, the Paf1C is minimally composed of Paf1, Ctr9, Cdc73, Leo1, and the higher eukaryote-specific subunit, Ski8, which is involved in mRNA surveillance [150–152]. A few reports differ on whether human Rtf1 is absent from [98, 150, 151] or present in [113] the human complex. Therefore, human Rtf1 appears to be less stably associated with the Paf1C. Consistent with this, Rtf1 is not stably associated with the *Drosophila* Paf1C [153]. However, despite its less stable association with the Paf1C, human Rtf1 still influences gene expression and histone modification [95, 154]. 

The Paf1C has evolved critical functions in higher eukaryotes, including promoting proper development, maintaining pluripotency in stem cells, and preventing cancer. Consistent with an important role in development, the human Paf1C is required for proper transcription of Wnt target genes [155] and *HOX* genes [98]. Additionally, Rtf1 regulates the transcription of Notch target genes in *Drosophila* and zebrafish [153, 156, 157]. Given the regulation of important developmental genes, it is not surprising that, in zebrafish, the Paf1C is required for the development of the ears, neural crest, and heart [156, 158]. For proper heart development, the Paf1C is critical for the specification of cardiomyocytes and patterning of the primitive heart [159]. In addition to genes required for proper development, the human Paf1C regulates the expression of interleukin-6 responsive inflammatory genes [160] and those that maintain pluripotency in stem cells [95]. 

Members of the human Paf1C have also been implicated in cancer. Pancreatic differentiation factor 2/Paf1 is overexpressed in pancreatic cancer cell lines and overexpression in cell culture results in transformation [161]. Additionally, the gene encoding human Paf1 is amplified in many cancers, including breast and uterine cancers [162, 163]. Furthermore, parafibromin/Cdc73 is a tumor suppressor encoded by HRPT2, a gene that is mutated in hyperparathyroidism-jaw tumor syndrome [164–166]. The roles of the Paf1C in preventing cancer are not entirely understood. However, the Paf1C promotes leukemogenesis through interactions with MLL-rearranged oncoproteins, a topic which has been recently reviewed [167]. 

Like the yeast counterpart, the human Paf1C influences gene expression by facilitating histone modifications. For example, the Paf1C, H2B monoubiquitylation, and H3 K4 and K79 methylation promote *HOX *gene expression [98]. H2B monoubiquitylation also appears to play a role in maintaining pluripotency in stem cells, and the Paf1C promotes the transcription of genes required for pluripotency in both mouse and human embryonic stem cells [95, 168]. Cell differentiation is associated with reduced expression of Paf1C subunits [95, 168] and reduced levels of H2B ubiquitylation [169]. Interestingly, the silencing of pluripotency genes upon differentiation may be accomplished by the interaction between the Paf1C and DNA methyltransferases [170], which repress these genes [171]. 

In addition to its effects on histone modifications, the human Paf1C regulates gene expression through direct interactions with gene-specific activators. Human Ctr9 associates with Stat3 and recruits it to the promoters of interleukin-6 responsive genes [160]. Cdc73 in humans promotes the transcription of Wnt target genes through a direct interaction with *β*-catenin [155]. Additionally, the Paf1C is found in a complex with the transactivator Tat to promote transcription from the HIV-1 promoter [172].

## 9. Conclusions and Future Studies

The Paf1C performs multiple functions during RNA pol II transcription, and these functions are conserved from yeast to humans. Previous studies have provided a wealth of knowledge about the roles of the Paf1C in transcriptional regulation; however, many important questions remain. While it is known that the Paf1C associates with RNA polymerase during elongation and dissociates near the poly(A) site, the details of the Paf1C-RNA pol II interaction, and its regulation, remain undefined. Current information suggests that the association of the Paf1C with RNA pol II is facilitated by multiple interactions. Cdc73 and Rtf1 play nonredundant roles in tethering the Paf1C to RNA pol II, but it is unclear whether these proteins make direct or indirect contacts with the polymerase or whether, like Leo1 [36], interactions with the nascent transcript are involved. Furthermore, although phosphorylation of Spt5 stimulates Paf1C recruitment [37–39], the regulatory events that promote dissociation of the Paf1C near the poly(A) site have yet to be elucidated.

A requirement for the Paf1C in regulating transcription termination and RNA 3′ end formation has been observed at specific genes. However, determining the scope of this effect on RNA 3′ end formation or other steps in RNA maturation will require additional genome-wide studies on Paf1C-deficient cells. Existing data indicate that the Paf1C mediates transcription termination in several ways. Its physical association with RNA processing factors suggests that the Paf1C coordinates the exchange of transcription elongation factors for transcription termination and 3′ end processing factors [31], although the mechanisms remain to be characterized. Additionally, the transcription termination functions of the Paf1C correlate with its roles in promoting serine 2 phosphorylation of the RNA pol II CTD [13, 31] and H2B ubiquitylation [73]. It is not known whether the Paf1C promotes phosphorylation of serine 2 by affecting the recruitment and/or activity of the CTD kinase, Ctk1. Furthermore, the mechanisms by which the Paf1C promotes histone modifications have not been thoroughly investigated. While the Paf1C facilitates the recruitment of histone modifying enzymes to ORFs [9–12, 70], the molecular details of these interactions are uncharacterized, and it is uncertain whether the Paf1C plays a role in histone modification beyond simply recruiting the active players. Finally, while both positive and negative effects on gene expression have been described for the Paf1C [14, 17, 92, 173] and its downstream histone modifications [88, 92], the features of a gene that confer Paf1C-dependent expression are unknown. In the case of the H2B ubiquitylation mark, it would be especially interesting to know why some genes are repressed by this modification, while others are activated by it [88, 92]. 

Although it has been shown that the Paf1C is required for proper expression of numerous genes throughout the yeast genome [14], a role in regulating the expression of noncoding RNAs (ncRNAs) has not been determined. In addition to genome-wide analyses unexpectedly localizing RNA pol II to intergenic regions [174], genome-wide transcription analyses have revealed that up to 85% of the yeast genome is transcribed [175, 176]. Similar results were obtained in human cells, such that ncRNAs account for a large portion of the transcription observed [175–178]. Many ncRNAs arise from start sites within intergenic regions and overlap with coding genes [175, 176]. Importantly, ncRNAs are becoming increasingly recognized as key regulators of gene expression. Therefore, to fully appreciate the mechanisms by which the Paf1C regulates gene expression, it will be important to know how its functions impact ncRNA synthesis. Upcoming investigations, which incorporate a multidisciplinary approach of structural, genetic, biochemical, and genomic experiments, will likely further establish the Paf1C as a critical regulator of gene expression, uncover new activities of the complex, and elucidate the molecular mechanisms of Paf1C-dependent functions that are crucial for the prevention of cancer and developmental defects.
